# Artificial Dentures

**Published:** 1860-04

**Authors:** John Allen


### ARTICLE IX.

### *Artificial Dentures.* By Dr. John Allen.

Lower sets of artificial teeth are often more difficult of adaptation than upper ones, owing to the great amount of absorption that may have taken place on the lower jaw, leaving scarcely any ridge or foundation for them to rest upon, and in some instances the place where the ridge ought to be, there is only a deep hollow, with muscles, ducts and glands, almost closing over it on either side. These tend to buoy up the denture and keep it afloat in the mouth, which renders it useless, if the plate is so wide as to rest upon them.

In order to overcome the difficulty when such cases occur, the impression should be taken with great care to prevent the inner folds of the cheek from overlaying the alveolar ridge, or hollow. These folds can be easily raised and moved outward with the fingers when the impression is being taken. After the impression has become hard, it should be trimmed so as form a very narrow bed, from which to form the plate. When the submaxillary ducts, sublingual glands, or their connecting fibrous tissues, raise above the hard ridge when the tongue or muscles are moved, the base should be so narrow as to allow them to pass up on the inside of the plate and teeth without dislodging the denture. For such cases the plate should be very thick and narrow, with smooth round edges, and the teeth should be quite thin.

### *How long, after the Extraction of Natural Teeth, before an Artificial Denture should be Inserted.*

From one to two weeks the writer deems sufficient time, under ordinary circumstances, for the patient to go without teeth; and in many instances even a shorter period would suffice.

But as there is a difference of opinion upon this point, we will present the different theories and submit to the reader their claims to supremacy. The objections urged to an immediate insertion of a denture are: that the gums will not become smooth and symmetrical if a plate be fitted to them before the alveolar processes have fully absorbed, but will conform in a greater or less degree to the irregularities of the temporary plate, and prevent a good practical result; consequently they advise their patrons to go without teeth at least one year.

This theory we think erroneous, because the slight undulating surface of the gums which the plate may have occasioned, presents no valid objection; for a permanent plate can be fitted to them just as perfectly at the expiration of one year, as if a temporary plate had not been worn, and the wearer will have become so well accustomed to artificial teeth, that the second set can be worn and used at once without difficulty, if properly constructed.

Again, the longer the natural teeth have been out, the harder it is for a person to acquire the use of artificial substitutes, for the lower jaw is thrown forward of its natural position, the lips become compressed, the muscles of the mouth and face contracted, and the tongue habituated to certain movements in munching food, which tend to dislodge the dentures when attempting to use them, and the original expression in many instances is lost.

The advantages of an immediate insertion of artificial teeth after the removal of the old ones are,

1st. The patient will acquire the faculty of using them satisfactorily in much less time than if required to go without any teeth until the healing of the gums and absorption of the alveolar processes have fully taken place.

2d. The original expression can be much better preserved, as the denture prevents that compression of the lips, and contraction of the muscles about the mouth and face, which the absence of the teeth will cause.

Temporary sets should be as skillfully made as permanent sets. There are many operators, however, who insert cheap and rude temporary sets of teeth, who seem to attach but very little importance as to how they are constructed for they are only intended to be worn a year or two, which is deemed a sufficient apology for poor work. The price usually paid for temporary teeth will not justify the use of the best materials, nor the highest degree of skill and taste in their construction. This is another reason for cheap work.

Here is an error that ought to be corrected, for in this way the patient has but a miserable substitute for the natural teeth, and is often subjected to extreme mortification at their rude and uncomely appearance and want of utility.

Again, the dentist often loses caste by this kind of work, for a poor, cheap specimen is often looked upon by others as a fair sample of the skill of him who made it. Although the inference may be very unjust, yet it tells against him. We think reform in this respect would prove mutually advantageous to the patient and operator.

Instead of this cheap graceless work, let the first set be as good as the second, or as near it as the case will admit, and let the charges be based upon terms of equity. Then the patron will be better served and better satisfied than with a rude fixture, devoid of the essential requisites of an artificial denture; for however perfectly formed and adapted artificial teeth may be, they usually meet with stern resistance when first inserted, (especially full sets,) for the tongue, muscles, glands and ducts, all conspire to eject them from the mouth as unwelcome intruders. They seem not to have forgotten the old offenders that used to create such painful sensations in their neighborhood until they were all exterminated, since which time there has been comparative peace.

But the sterner will can subjugate all these opposing forces and establish harmony, provided the dentures are rightly formed, which is the condition of the compromise. The tongue will then acquire more cautious habits, and avoid those movements which at first would send them whirling out of the mouth, for the tongue is the first to repel invasion, it does not like to be restrained. The contiguous muscles, ducts and glands, also require freedom of action and they must not be infringed, for this is nature's law. But if their natural movements and functions are provided for, then the teeth will be permitted to remain quietly in their proper places and subserve the purposes for which they were designed.

But still, even with the most perfect set of artificial teeth there will be in many instances a stiff and restrained feeling and appearance about the mouth, when first inserted, that time only can remove, especially if the natural teeth have long been out. Patience and perseverance will, however, entirely overcome the difficulties that at first appeared so formidable to a new beginner.

(To be continued.)

